# Well-differentiated abdominal liposarcoma: experience of a tertiary care center

**DOI:** 10.1186/s12957-015-0580-z

**Published:** 2015-05-01

**Authors:** Kursat Karadayi, Caglar Yildiz, Savas Karakus, Atilla Kurt, Birkan Bozkurt, Sinan Soylu, Ayse A Cicekli, Reyhan Egilmez, Ali Cetin

**Affiliations:** Department of Surgery, Faculty of Medicine, Cumhuriyet University, Sivas, 58140 Turkey; Department of Obstetrics and Gynecology, Faculty of Medicine, Cumhuriyet University, Sivas, 58140 Turkey; Department of Pathology, Faculty of Medicine, Cumhuriyet University, Sivas, 58140 Turkey; The Division of Surgical Oncology, Faculty of Medicine, Cumhuriyet University, Sivas, 58140 Turkey

**Keywords:** Liposarcoma, Abdomen, Surgical management

## Abstract

**Background:**

We presented abdominal liposarcoma cases diagnosed and managed in a tertiary care center and also conducted a literature review on main features of this tumor.

**Methods:**

Chart reviews of eight cases were conducted, and clinical, surgical, histopathological, and follow-up data were recorded.

**Results:**

Overall, complete surgical resection was performed with adjacent organ resection in 25% of cases, and radiotherapy was not administered. Recurrence was developed in only one case and died after 2 years and 3 months, and other cases are under follow-up without recurrence. Histopatological examinations revealed findings of well-differentiated liposarcoma.

**Conclusions:**

According to our surgical experience, the surgical margin positivity may not be a determining factor for the survival of patients with well-differentiated liposarcoma, and in the absence of macroscopic invasion, adjacent organ resection may not be required. Radiotherapy may not be preferred when complete resection of abdominal mass was achieved.

## Background

Liposarcoma of abdomen is a rare lesion consisted of malign fat cells and accounts for approximately 20% of all mesenchymal malignancies in adults. These lesions may be found in different organs, typically occurs in either the retroperitoneum or the extremities [1]. Abdominal liposarcomas are generally located in retroperitoneum; it is often difficult to collect pathological samples nonsurgically. Surgical exploration is needed for the final pathologic diagnosis. Retroperitoneal sarcomas tend to be high grade, except liposarcomas, they tend to be low to intermediate grade. Histologic grade is the main factor for survival rates in patients with liposarcomas [2]. Symptoms are usually nonspecific, and they do not appear until the tumor becomes very large, a painless abdominal mass that enlarges in a long period of time in an adult is most common history of the patients. Metastases at the time of initial presentation are uncommon [3]. Surgical treatment is the main modality in the therapy of retroperitoneal liposarcomas [3]. However, to our best knowledge, there is a small number of case report or series related to abdominal liposarcoma. The knowledge related to the clinical features and course of retroperitoneal liposarcoma is mainly limited single-institutional experiences. We presented cases with abdominal liposarcoma managed in a tertiary care center and also conducted a literature review on presentation, management, and outcomes of these patients.

## Methods

**Figure 1:**
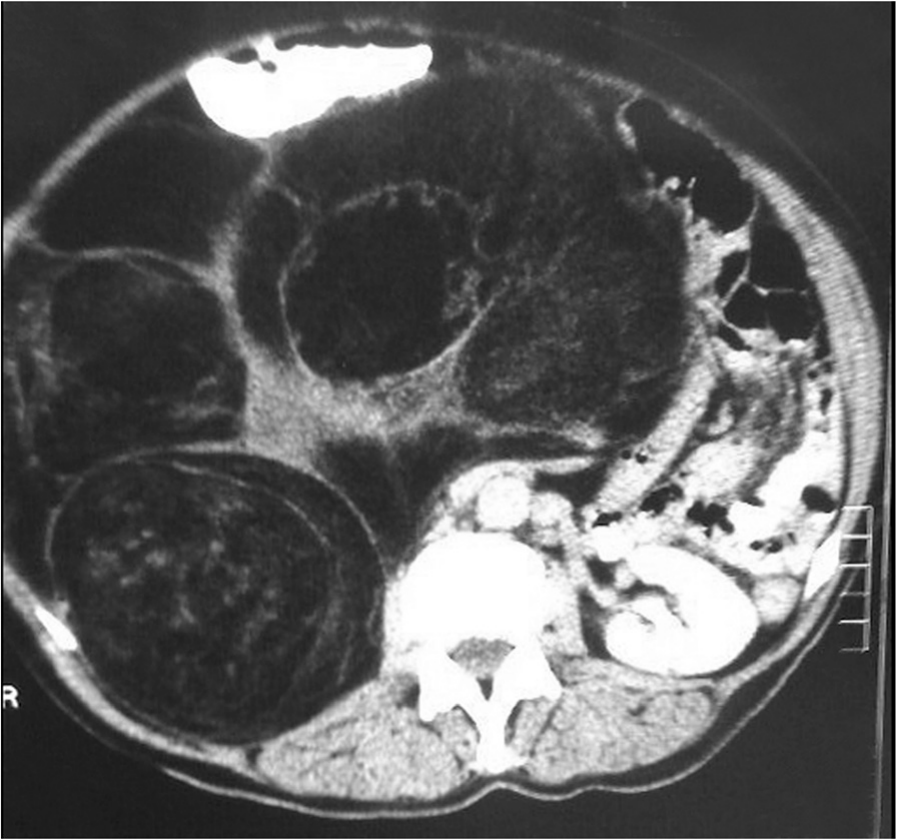
Computerized tomography was used for imaging of abdomen in all patients.

**Figure 2:**
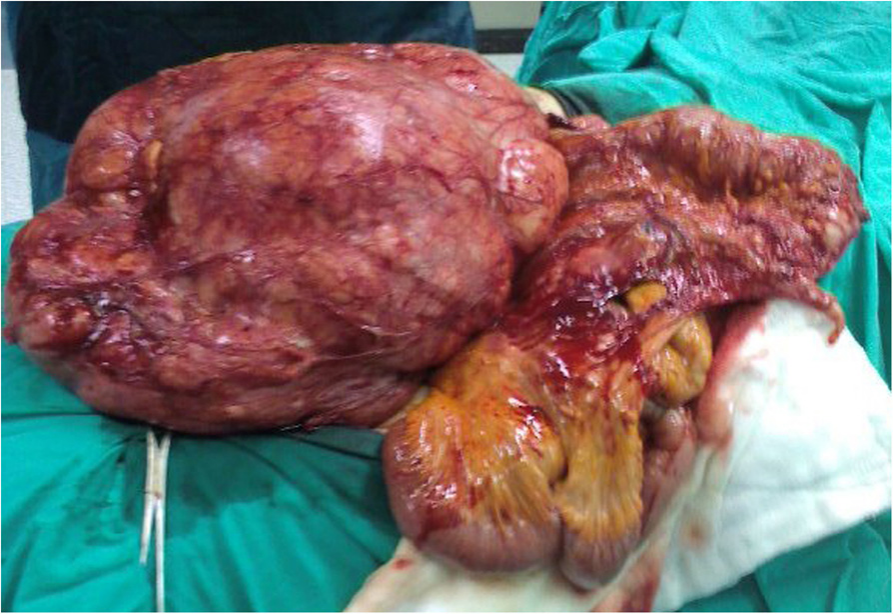
A midline abdominal incision dissection of the mass.

**Figure 3:**
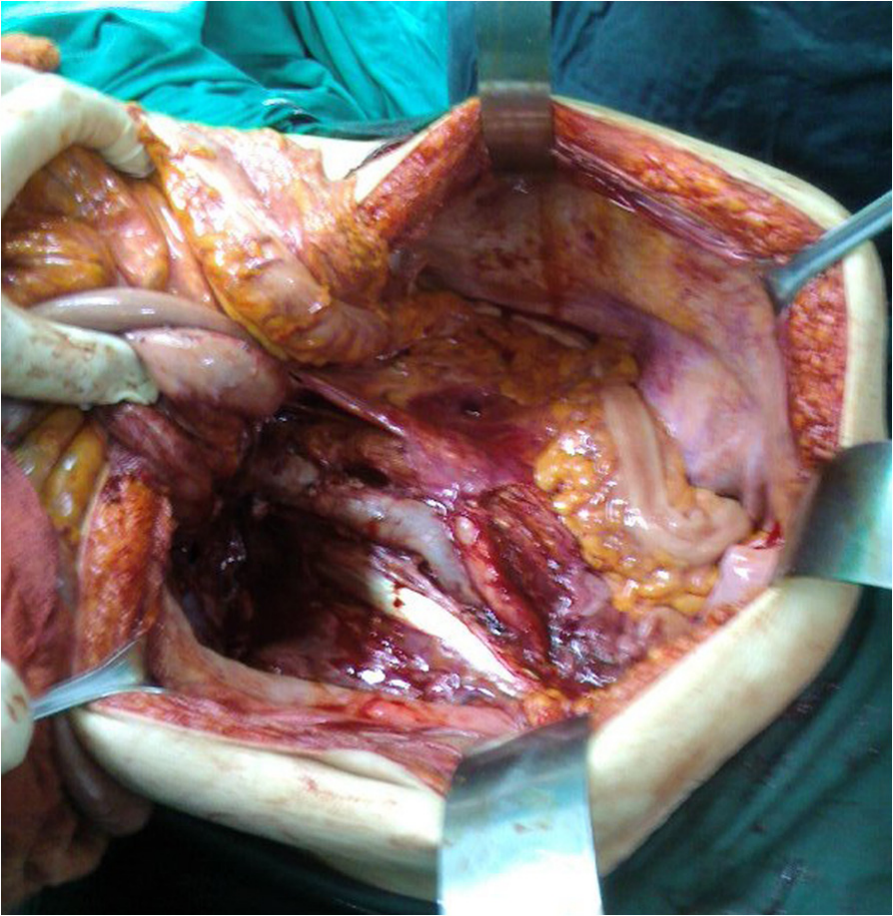
Sharp and blunt dissection of the mass.

**Figure 4:**
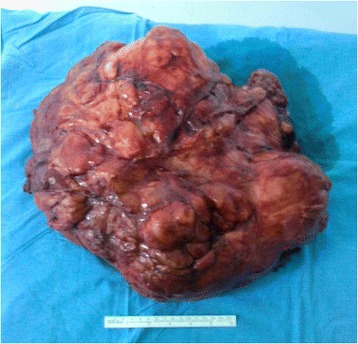
The organs that involved macroscopically by the tumor were excised.

Chart reviews of eight cases managed with liposarcoma at the General Surgery Service of our tertiary care university hospital were conducted between December 2011 and January 2014. This study was approved by the Human Ethics Committee of our university. Clinical information regarding the age, gender, clinical findings, imaging findings, surgical procedure, histopathology of the tumor, follow-up findings, overall survival were recorded. Any imaging studies that had been obtained preoperatively were also reviewed. Computerized tomography was used for imaging of abdomen in all patients (Figure 1). A midline abdominal incision and sharp and blunt dissection of the mass (Figures 2 and 3) was performed and then intraoperative frozen section technique was used to evaluate the margins of the tumoral mass for all cases. The organs that involved macroscopically by the tumor were excised (Figure 4). Tissue specimens were processed by the hematoxylin-eosin staining method.

All the patients were followed up at 1, 3, 6, and 12 months and 6-month period in second year. Physical and ultrasonographic examinations with laboratory tests were performed in the third month, and a computerized tomography was performed in the sixth month after surgery. The median follow-up of 21 months (min.15, max.24) with the shortest follow-up of 15 months and longest follow-up of 24 months.

## Results

**Figure 5:**
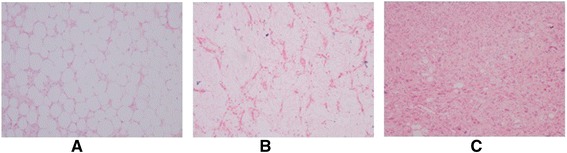
Histopatological examination of mixed-type liposarcoma tissue in case 4. **(A)** Well-differentiated component (HEX100). **(B)** Myxoid component (HEX100). **(C)** Undifferentiated component (HEX100).

Table 1 presents selected demographic, clinical, and intraoperative features of cases. Of eight patients, four patients were female and median age was 61.5 (21 to 73). Overall, the most important clinical finding is palpable abdominal mass and abdominal pain. During computed tomography (CT) examination, we observed a large abdominal mass about 28 to 50 cm in the largest size with heterogeneous structure displacing adjacent organ and tissues. Complete resection of mass was adequate as surgical management in cases 1, 3, 5 to 8. In case 2, right nephrectomy and right ureter resection were performed due to tumoral invasion, and a second surgery was also required for local recurrence; however, histopathological diagnosis was the same with other cases except case 4. In case 4, right hemicolectomy, ileal resection, and partial bladder resection were added due to local invasion, and histopathological diagnosis was liposarcoma, well-differentiated, and dedifferentiated (mixed) (Figure 5). The blood loss during surgery was acceptable (150 to 220 mL), mean time for surgical procedure was 140 min, and the duration of hospital stay ranged from 4 to 6 days.

**Table 1:** **Patient demographics and clinical and intraoperative findings**

**Patient number**	**Age (y)**	**Gender**	**Clinical findings**	**CT imaging findings**	**Surgical procedures**	**Survival after surgery**
Case 1	43	F	Palpable mass at left lower quadrant	A 16 × 10 × 18-cm heterogeneous pelvic mass that extends up to the right lower quadrant of the abdomen.	Complete resection of mass without additional organ resection	Alive 21 months
Case 2	63	F	Palpable mass of abdomen, general abdominal pain	A 50 × 40 × 28-cm heterogeneous abdominal mass compressing aorta and vena cava inferior.	During first surgery, complete resection of mass with additional right nephrectomy and right ureter resection.	Recurrence 2 years later after first surgery and died due to renal failure 3 months later after second surgery
					During second surgery performed for local recurrence 2 years later, right hemicolectomy, cholecystectomy, ileal resection.	
Case 3	73	F	General abdominal pain	A 21 × 15 × 5-cm heterogeneous retroperitoneal mass.	Complete resection of mass without additional organ resection	Alive 15 months
Case 4	61	M	Palpable mass of abdomen, general abdominal pain	A 15 × 8 × 11-cm heterogeneous retroperitoneal mass.	Right hemicolectomy, ileal resection, partial bladder resection	Alive 20 months
Case 5	28	M	General abdominal pain, abdominal swelling	A 30 × 18 × 27-cm heterogeneous septated abdominal mass that displaces the adjacent organs.	Complete resection of mass without additional organ resection	Alive 24 months
Case 6	62	F	Upper abdominal pain	A 19 × 10 × 18-cm heterogeneous pelvic mass that extends above the umbilicus.	Complete resection of mass without additional organ resection	Alive 22 months
Case 7	66	M	General abdominal pain, abdominal swelling	A 24 × 20 × 12-cm heterogeneous retroperitoneal mass.	Complete resection of mass without additional organ resection	Alive 21 months
Case 8	21	M	General abdominal pain, abdominal swelling	A 23 × 10 × 15-cm cystic septated abdominal mass at right lower quadrant of the abdomen.	Complete resection of mass without additional organ resection	Alive 15 months

Y, year; F, female; M, male.

In almost all cases, histopatological examinations revealed findings of well-differentiated and grade 1 liposarcoma. Surgical margins were defined as positive in 75% of cases and necrosis was detected in 25% of cases.

Recurrence has occurred in only one case and died due to renal failure. Intraoperatively, in case 2, vena cava inferior injury was developed and repaired. Four patients had minor complication according to Clavien-Dindo classification [4].

## Discussion

Eight patients with abdominal liposarcoma were presented in this case series. Overall, complete surgical resection was achieved with adjacent organ resection in 25% of cases. We observed recurrence in only one case who died within 2 years and 3 months, and other cases are under follow-up without recurrence. After median follow-up of 21 months (min.15, max.24), all cases were alive without recurrence except case 2 who survived 2 years and 3 months after first surgery. Although radiotherapy was not administered to our cases, there was no recurrence except case 2.

Liposarcoma is the most common mesenchymal tumor of the retroperitoneal space but continues to pose a challenge with regard to diagnosis, prediction of clinical behavior, and treatment of disease recurrence within the abdominal/retroperitoneal space [5]. The major problem of soft tissue sarcomas being present in the extremities like retroperitoneum, chest wall, head and neck, and subcutaneous tissues is the other sites can be affected. Sarcomas are a rare and heterogeneous group of malignant tumors of mesenchymal origin that comprise all adult and childhood malignancies, respectively 1% and 12% [6,7]. Sarcomas constitutes one third of malignant tumors that arise in the retroperitoneum, and approximately 10% to 15% of soft tissue sarcomas arise in the retroperitoneum [8,9]. Mesenchymal cells of muscle, fat, and connective tissues are the origins of sarcomas that arise from retroperitoneum.

The most commonly encountered histologic subtypes of retroperitoneal sarcoma are liposarcoma (41%), leiomyosarcoma (28%), malignant fibrous histiocytoma (7%), fibrosarcoma (6%), and malignant peripheral nerve sheath tumor (3%) [10]. Liposarcoma occurs in three main biologic forms: well-differentiated liposarcoma, myxoid and/or round cell, and pleomorphic. In rare circumstances, lesions can have a combination of morphologic types; these are classified as combined or mixed-type liposarcomas. The most recent World Health Organization classification of soft tissue tumors recognizes five categories of liposarcomas: well differentiated, which includes the adipocytic, sclerosing, and inflammatory subtypes; dedifferentiated; myxoid; round cell; and pleomorphic [11,12].

Well-differentiated (low-grade) liposarcomas are the most common types of liposarcomas, followed by dedifferentiated liposarcomas. The amount of lipid inside the cells, the mucoid lipid, and the degree of cell differentiation are the essential of the classification. Myxoid, round cell, and pleomorphic liposarcomas are rare in the retroperitoneum. Myxoid and round cell liposarcomas share the same reciprocal translocation t(12,16)(q13; p11), in which the *CHOP* gene is inserted adjacent to a novel gene called *FUS* or *TLS* (translocated in liposarcoma). While no specific chromosomal translocations have been identified in well differentiated/dedifferentiated liposarcomas, amplification of *MDM2* and *CDK4* is very frequent in these subtypes, and their identification may be useful diagnostically. Furthermore, overexpression of *MDM2* and *CDK4* is also being exploited for therapeutic gain [12,13].

Liposarcomas are often asymptomatic until they reach to large size in a long period of time before producing any symptoms, and the complaints of patients are mainly related to direct invasion or compression of other adjacent organs. There are no significant laboratory abnormalities in the earlier stages and have often grown to a large size by the time they are identified using a diagnostic modality such as US or CT. CT of the abdomen is the most useful tool in the imaging of retroperitoneum. CT of the chest is very important for evaluating the lungs, as they are the first site of metastasis in the most of cases. A CT scan allows not only assessment of the tumor’s location and its relationship to adjacent organs but also identification of metastatic lesions in the liver or peritoneal cavity [14]. CT is less sensitive to motion artifact than magnetic resonance image (MRI); because of this property, CT defines the anatomic relationship of the tumor to other abdominal organs better than the MRI. Liposarcoma of the abdominal region, although rare, needs to be differentially diagnosed from other abdominal tumors. Its symptoms are nonspecific, and its diagnosis is intriguing. CT-scan or MRI are the most useful tools for investigation and evaluation of retroperitoneal mass [14]. Surgery is the mainstay of treatment for these cases and is curative with no recurrence following complete surgical excision [15]. The final diagnosis should always be confirmed with histopathology of the specimen. We think that although in limited number, these described cases can contribute to the great amount of controversy regarding surgical management of abdominal liposarcoma.

Surgery is indispensable for treatment of abdominal liposarcoma. Complete surgical removal of retroperitoneal tumor is the most effective treatment and has a significant effect on the survival rate. The primary aim of the surgery is complete resection with negative margins. However, in many cases, it is not possible to perform complete resection because of the tumor being too large or invasive to organs around it or its relation with the great vessels.

There is continuing research and debate on the use of intraoperative radiotherapy (RT), adjuvant RT, preoperative RT, preoperative intensity-modulated RT (IMRT), preoperative chemoradiotherapy, preoperative chemotherapy, and adjuvant chemotherapy. Many studies have shown that a significant number of patients experience prolonged disease-free survival when all grossly evident recurrent disease can be resected. Chemotherapy or radiotherapy administration is still controversial in the treatment of locally recurrent disease [14]. A phase III randomized controlled trial has been completed yet about radiotherapy in patients with primary soft tissue sarcoma of the retroperitoneum or pelvis [16].

Because of the huge sizes of most retroperitoneal sarcomas, the size of the tumor is not a predictor for survival of the disease. Tumor grade has been reported as a significant factor in some studies, with the weight of evidence supporting shorter recurrence-free and overall survival for patients with high-grade tumors [17,18]. Metastasis potential of liposarcomas is very low; because of their tendency for local recurrence in the retroperitoneum/mediastinum and spermatic cord, these same tumors are referred to as well-differentiated liposarcomas in these locations [7,19]. Well-differentiated liposarcomas are low-grade tumors; compared to dedifferentiated high-grade liposarcomas, they have lower recurrence rates, the potential to metastasize, and dedifferentiated liposarcomas have a six-fold higher risk of death [5,19].

Overall, 5-year survival for well-differentiated subtypes is 90%, while 5-year survival for pleomorphic subtypes is only 30% to 50%. Dedifferentiated and myxoid/round cell subtypes have 5-year survival rates of 75% and 60% to 90%, respectively [2]. Well-differentiated liposarcomas may recur locally, but metastatic potential is low compared with pleomorphic liposarcomas that have high metastatic potential, and pleomorphic liposarcomas have shorter survival than well-differentiated liposarcomas [5].

## Conclusions

In summary, abdominal liposarcomas are low-grade tumors in general and surgical excision with wide margin is decreasing the effectiveness of the radiotherapy, increasing the adverse effects especially on intestines. In our cases, although surgical margin was reported as positive in 75% of patients, because of the absence of macroscopic invasion, adjacent organ resection was not performed. According to our surgical experience, in patients with retroperitoneal liposarcoma, surgical resection can be completed considerably easy than expected although its size can be huge. In almost all cases, absence of invasion to adjacent tissues and organs is another advantage during surgical management, adjacent organ resection should not be necessary because of the low invasion potential of these tumors.
